# Apiaceae Essential Oils: Boosters of Terbinafine Activity against Dermatophytes and Potent Anti-Inflammatory Effectors

**DOI:** 10.3390/plants10112378

**Published:** 2021-11-04

**Authors:** Adriana Trifan, Simon Vlad Luca, Andra-Cristina Bostănaru, Mihai Brebu, Alexandra Jităreanu, Romeo-Teodor Cristina, Krystyna Skalicka-Woźniak, Sebastian Granica, Monika E. Czerwińska, Aleksandra Kruk, Hélène Greige-Gerges, Elwira Sieniawska, Mihai Mareș

**Affiliations:** 1 Department of Pharmacognosy, Faculty of Pharmacy, Grigore T. Popa University of Medicine and Pharmacy Iasi, 700115 Iasi, Romania; 2 Biothermodynamics, TUM School of Life and Food Sciences, Technical University of Munich, 85354 Freising, Germany; vlad.luca@tum.de; 3 Laboratory of Antimicrobial Chemotherapy, Faculty of Veterinary Medicine, “Ion Ionescu de la Brad” Iasi University of Life Sciences, 700489 Iasi, Romania; mihaimares@fungi.ro; 4 Physical Chemistry of Polymers Laboratory, Petru Poni Institute of Macromolecular Chemistry, 700481 Iasi, Romania; bmihai@icmpp.ro; 5 Department of Toxicology, Faculty of Pharmacy, Grigore T. Popa University of Medicine and Pharmacy Iasi, 700115 Iasi, Romania; alexandra.jitareanu@umfiasi.ro; 6 Department of Pharmacology, The Banat University of Agricultural Sciences and Veterinary Medicine, 300645 Timisoara, Romania; rtcristina@yahoo.com; 7 Department of Natural Products Chemistry, Medical University of Lublin, 20-093 Lublin, Poland; kskalicka@pharmacognosy.org (K.S.-W.); esieniawska@pharmacognosy.org (E.S.); 8 Microbiota Lab, Centre for Preclinical Studies, Department of Pharmacognosy and Molecular Basis of Phytotherapy, Medical University of Warsaw, 02-097 Warsaw, Poland; sgranica@wum.edu.pl (S.G.); akruk@wum.edu.pl (A.K.); 9 Department of Biochemistry and Pharmacogenomics, Faculty of Pharmacy, Medical University of Warsaw, 02-097 Warsaw, Poland; monika.czerwinska@wum.edu.pl; 10 Centre for Preclinical Research, Medical University of Warsaw, 02-097 Warsaw, Poland; 11 Bioactive Molecules Research Laboratory, Department of Chemistry and Biochemistry, Faculty of Sciences, Section II, Lebanese University, Jdaidet el-Matn B.P. 90656, Lebanon; helenegreige73@gmail.com

**Keywords:** essential oil, *Trichophyton rubrum*, checkerboard assay, *Coriandrum sativum*, cytokines, *Trachyspermum ammi*, terbinafine, *Carum carvi*, synergy, *Pimpinella anisum*

## Abstract

Dermatophyte infections represent an important public health concern, affecting up to 25% of the world’s population. *Trichophyton rubrum* and *T. mentagrophytes* are the predominant dermatophytes in cutaneous infections, with a prevalence accounting for 70% of dermatophytoses. Although terbinafine represents the preferred treatment, its clinical use is hampered by side effects, drug–drug interactions, and the emergence of resistant clinical isolates. Combination therapy, associating terbinafine and essential oils (EOs), represents a promising strategy in the treatment of dermatophytosis. In this study, we screened the potential of selected Apiaceae EOs (ajowan, coriander, caraway, and anise) to improve the antifungal activity of terbinafine against *T. rubrum* ATCC 28188 and *T. mentagrophytes* ATCC 9533. The chemical profile of EOs was analyzed by gas chromatography. The minimal inhibitory concentration (MIC) and minimal fungicidal concentration (MFC) of EOs/main compounds were determined according to EUCAST-AFST guidelines, with minor modifications. The checkerboard microtiter method was used to identify putative synergistic combinations of EOs/main constituents with terbinafine. The influence of EOs on the viability and pro-inflammatory cytokine production (IL-1*β*, IL-8 and TNF-*α*) was determined using an ex vivo human neutrophils model. The binary associations of tested EOs with terbinafine were found to be synergistic against *T. rubrum*, with FICI values of 0.26–0.31. At the tested concentrations (6.25–25 mg/L), EOs did not exert cytotoxic effects towards human neutrophils. Anise EO was the most potent inhibitor of IL-1*β* release (46.49% inhibition at 25 mg/L), while coriander EO displayed the highest inhibition towards IL-8 and TNF-*α* production (54.15% and 54.91%, respectively). In conclusion, the synergistic combinations of terbinafine and investigated Apiaceae EOs could be a starting point in the development of novel topical therapies against *T. rubrum*-related dermatophytosis.

## 1. Introduction

Dermatophytes are filamentous fungi with a high affinity for the keratinized tissue of the skin, nails, and hair, causing superficial infections with different degrees of inflammation known as dermatophytoses [1,2]. According to data from the World Health Organization, dermatophytosis has become a public health issue, affecting up to a quarter of the global population; its incidence is influenced by different factors, e.g., age, sex, the season, socioeconomic status, and geographical region [3]. *Trichophyton* species are the main causative agents of dermatophytosis, accounting for up to 70% of total cases; *Trichophyton rubrum* is the major etiological agent of the cutaneous infection of the feet, nails, and body, followed by *T. mentagrophytes* [4,5].

Terbinafine, a synthetic allylamine derivative, is currently the “golden standard” treatment in dermatophytosis and acts as a potent inhibitor of the fungal squalene epoxidase, an enzyme involved in ergosterol biosynthesis [6]. Still, its oral use is hampered by common side effects such as gastrointestinal complaints, rash, headache, and loss of taste. The emergence of recalcitrant dermatophytoses that require a long-term treatment regimen can lead to severe hepatotoxicity, making the monitoring of the liver function mandatory [6,7,8]. Moreover, in numerous cases, systemic therapy is not recommended due to putative drug–drug interactions and patient co-morbidities [9]. In addition, the topical use of terbinafine in onychomycosis is hampered by its poor nail penetration and low efficacy, as revealed in several clinical trials, which evaluated the potency of terbinafine nail solutions and sprays [10,11]. Even though, in the late 2010s, terbinafine-resistant dermatophytes were seldomly reported, nowadays an alarming number of clinical isolates that are resistant to terbinafine have emerged as a consequence of misused or repeated topical and systemic treatments. Thus, dermatophytosis cases have become refractory to antifungal drugs, with frequent relapses and the chronicity of fungal infection [6,12,13].

Combinatorial strategies associating conventional antifungals with plant-derived products represent a promising approach to increase the efficacy of extant treatment regimens in dermatophytosis and to overcome fungal resistance [14,15]. In this context, synergistic combinations can exert a multi-target antifungal activity, thus increasing the potency of conventional drugs and tackling multi-drug microbial resistance. In addition, such associations have the advantage of reducing the effective doses of conventional drugs, thus minimizing their toxicity and side effects [16,17]. Among plant-derived products, essential oils (EOs) possess valuable biological properties and their potent and broad-spectrum antimicrobial activities generate impressive reports in the scientific literature [18,19,20]. Distillation or pressing are physical processes used to obtain EOs, which comprise a blend of small volatile molecules that act as signaling agents in plants (e.g., attraction of pollinators, antifeedant agents, and protection against microorganisms) [21,22]. The antidermatophytic propensities of EOs are also well-documented and include the disruption of the cell wall/membrane; the inhibition of spore germination, ergosterol and cellular proteins synthesis; efflux pumps, and biofilm formation [23,24]. Moreover, EOs exhibit anti-inflammatory effects, thus supporting lesion healing and alleviating symptoms related to dermatophytosis [14,15].

Apiaceae (Umbelliferae) family comprises 446 genera of 3540 herbaceous species distributed worldwide, many of them used in the food, pharmaceutical, and cosmetic industries [25,26]. A characteristic feature of Apiaceae members is the presence of volatile compounds which are found both in aerial parts and the underground systems of plants [27]. Apiaceae-derived essential oils are rich sources of monoterpenes, sesquiterpenes, and phenylpropanoids endowed with significant antibacterial, antifungal, and anti-inflammatory activities [28,29,30,31].

Among Apiaceae species, ajowan (*Trachyspermum ammi* L.), coriander (*Coriandrum sativum* L.), caraway (*Carum carvi* L.), and anise (*Pimpinella anisum* L.) are widely used as spices and flavoring agents and are also employed as remedies to treat various human ailments [20,27,29,31,32,33,34,35,36]. Their fruits are rich sources of EOs endowed with significant antibacterial and antifungal properties against a broad spectrum of microorganisms, including dermatophytes [20,29,31,37,38,39,40].

The treatment failure and resistance to terbinafine are key elements in driving research into alternative therapies for dermatophyte infections. As mentioned above, the effective strategies to treat recalcitrant infections include combination therapy. Considering the known antidermatophytic effects of selected Apiaceae species, the association with terbinafine might display a multi-target activity, increasing its potency and tackling fungal resistance. Hence, the aim of the present study was to assess, for the first time, the potential of EOs derived from the fruits of ajowan, coriander, caraway, anise, and their main constituents, and to enhance the terbinafine activity against *T. rubrum* and *T. mentagrophytes*. In addition, the influence of EOs on the viability and pro-inflammatory cytokine production in an ex vivo human neutrophils model was evaluated.

## 2. Materials and Methods

### 2.1. Chemicals

The following chemicals were purchased from Sigma-Aldrich (Steinheim, Germany): 4-(2-hydroxyethyl)-1-piperazineethanesulfonic acid) (HEPES), 3-(N-morpholino) propanesulphonic acid (MOPS), anethole, anhydrous glucose, carvone, chloramphenicol, cycloheximide, dimethyl sulfoxide (DMSO), dextran, fetal bovine serum (FBS), L-glutamine, linalool, limonene, propidium iodide, RPMI 1640 medium, terbinafine hydrochloride, thymol and urolithin A. Phosphate-buffered saline (PBS) was bought from Gibco (Hong Kong, China). The (Ca^2+^)-free PBS was acquired from Biomed (Lublin, Poland). The Human Pancoll (1.077 g/mL) was from PAA Laboratories (Pasching, Austria). Penicillin-streptomycin was purchased from Biowest (Nauillé, France). Lipopolysaccharide (LPS) from *Escherichia coli* 0111:B4 was purchased from Merck (Kenilworth, NJ, USA). Human ELISA sets (IL-1*β*, IL-8, TNF-*α*) were acquired from BD Biosciences (Franklin Lake, NJ, USA). Sabouraud dextrose agar (SDA) was bought from Biolab (Budapest, Hungary).

### 2.2. Plant Material

Dried plant materials (fruits of ajowan, coriander, caraway, and anise) were purchased from local pharmacies and their botanical identity was confirmed by one of the authors (A.T.). Voucher specimens (AJ 1/2019, CO 2/2019, CA 3/2019, AN 4/2019) were deposited in the Department of Pharmacognosy, Faculty of Pharmacy, “Grigore T. Popa” University of Medicine and Pharmacy Iasi, Romania.

### 2.3. Essential Oil Extraction

The powdered plant materials (100 g each) were subjected to hydrodistillation for 3 h in a Clevenger apparatus. EOs were dried over anhydrous sodium sulfate and stored in dark glass tubes at a temperature of 4 °C until further analysis.

### 2.4. Essential Oils Analysis

EOs analysis was undertaken by gas chromatography (GC) using an Agilent 6890N gas chromatograph coupled with a mass spectrometer (MS) detector (Agilent model 5975 inert XL) and a flame ionization detector (FID). TRB-5MS capillary columns (30 m length, 0.25 mm internal diameter, 0.25 μm film thickness) were used for each detector. The GC-MS conditions were as follows: injection volume of 0.2 μL, EOs samples being injected in the split mode (1:200); helium as carrier gas, with a flow rate of 0.5 mL/min; injector/detector temperature of 230 °C. Oven temperature was raised from 60 °C to 220 °C at a rate of 4 °C/min, and then to 320 °C at 20 °C/min; the final temperature was isothermally held for 7.5 min. MS was operated in full scan mode, with an ionization energy of 70 eV, and mass spectra were recorded in the range *m/z* 15–450. GC-FID conditions were similar to those used in GC-MS analysis, except for the carrier gas flow that was equal to 1.8 mL/min, and the splitting ratio (1:100). Retention indices were calculated for individual compounds using a standard solution of *n*-alkanes C8–C20 (Sigma-Aldrich). EOs constituents were identified by comparing their mass spectra with those from the NIST 11 mass spectra library, accepting only the quality of recognition above 80%, and by comparing their retention indices with those from the literature [41,42]. The relative percentages of individual constituents were obtained from the FID peak areas without correction factors.

### 2.5. Microbial Strains

The antifungal activities of the EOs (ajowan, coriander, caraway, and anise), their main constituents (thymol, linalool, carvone, limonene, and anethole), and terbinafine were investigated against standard strains from the American Type Culture Collection (*Trichophyton rubrum* ATCC 28188, *Trichophyton mentagrophytes* ATCC 9533). Prior to testing, fungi were grown on SDA and incubated up to 15 days at 30 °C. All strains were checked during incubation, and they were used when a maximal number of conidia were formed.

### 2.6. Antifungal Susceptibility Testing

The MIC values were assessed by the broth microdilution method using 96-well plates, according to The European Committee on Antimicrobial Susceptibility Testing Subcommittee on Antifungal Susceptibility Testing (EUCAST-AFST) guidelines [43] and following the methodology described by Saunte et al. [12] and Markantonatou et al. [44], with slight modifications. Serial double dilutions of EOs and volatile compounds (thymol, linalool, carvone, limonene, anethole), ranging from 4 to 2048 mg/L, were prepared in a RPMI 1640 medium 2% glucose buffered with 0.165 M MOPS, and supplemented with chloramphenicol 50 mg/L and cycloheximide 300 mg/L, followed by inoculation (10^5^ CFU/mL). Final concentration of DMSO did not exceed 1%. Terbinafine was used as reference drug (concentration range 0.015–8 mg/L). Fungi growth control and sterility control were used in order to assure the reliability of the tests. The incubation was performed at 30 °C for 7 days. The MIC was considered as the lowest concentration with no visual growth of the tested fungi. The MFC was assessed by inoculating 10 µL from wells that did not show growth in the MIC assay onto SDA plates, followed by incubation at 30 °C for 72 h. The MFC was considered as the lowest concentration with no growth or less than five colonies in the case of subculture (corresponding to ~99.9% killing activity). Experiments were run in triplicate.

### 2.7. Checkerboard Assay

The checkerboard broth microdilution method was used to evaluate putative interactions between EOs/volatile compounds (thymol, linalool, carvone, limonene, anethole) and terbinafine against *T. rubrum* and *T. mentagrophytes*. Serial double dilutions of EOs, volatile compounds and terbinafine were prepared as previously described for determination of MIC. EOs/volatile compounds were dispensed in increasing concentrations along the *X*-axis (1/128xMIC–4xMIC), while terbinafine was dispensed in increasing concentrations along the *Y*-axis (1/16xMIC–4xMIC), followed by inoculation (10^5^ CFU/mL) [12,45]. The plates were incubated at 30 °C for 7 days. Assays were performed in duplicate and repeated at least three times. The interactions between EOs/volatile compounds and terbinafine were interpreted based on the fractional inhibitory concentration index (FICI) values determined using the following equation:

FICI = FIC_EO/compound_ + FIC_terbinafine_

where:

FIC_EO/compound_ = MIC_EO/compound in combination_/MIC_EO/compound alone_

and:

FIC_terbinafine_ = MIC_terbinafine in combination_/MIC_terbinafine alone_

Synergy was defined by FICI ≤ 0.5: addition was considered when 0.5 > FICI ≤ 1; was indifference considered if 1 > FICI ≤ 4 and antagonism for FICI > 4 [17,22].

### 2.8. Evaluation of Cytokine Production in Human Neutrophils

#### 2.8.1. Neutrophils Isolation

The protocol used to prepare the buffy coat was in accordance with the principles stated in the Declaration of Helsinki; peripheral venous blood was collected from healthy male volunteers (ages 20–35 years) at the Warsaw Regional Blood Centre. Human neutrophils were isolated by dextran sedimentation and Pancol centrifugation [46], allowing a neutrophil preparation with over 97% purity. Neutrophils were suspended in PBS and kept at a temperature of 4 °C until subsequent analysis.

#### 2.8.2. Evaluation of Neutrophils Viability

Flow cytometry using propidium iodide (PI) staining was undertaken to evaluate the influence of Apiaceae EOs on neutrophils viability, following a previously described method [47]. Briefly, neutrophils (2.0 × 10^6^/mL) were cultured for 24 h in RPMI 1640 medium (supplemented with 10% FBS, 2 mM L-glutamine and 10 mM HEPES) in the absence/presence of EOs (6.25–25 mg/L) added 30 min before stimulation with 100 ng/mL LPS. After incubation, cells were centrifuged, washed, and re-suspended in PI solution 0.5 μg/mL, followed by incubation in the dark at room temperature (15 min). The neutrophils were analyzed using a BD FACSCalibur flow cytometer (BD Biosciences, San Jose, CA, USA) by recording 10.000 events for each sample. Cells showing high permeability to PI were considered PI+ cells. Neutrophils viability was calculated using the following formula: 100% − PI+ cells%. Triton X-100 (0.1%) was used as positive control.

#### 2.8.3. Evaluation of IL-1β, IL-8 and TNF-α Secretion

The evaluation of cytokine release from LPS-stimulated neutrophils was carried out following a protocol previously described [47]. Thus, neutrophils were incubated at 37 °C in a 96 well-plate in RPMI 1640 medium (prepared as described in Section 2.8.2), with/without the tested EOs (concentrations of 6.25, 12.5, and 25 mg/L) added 30 min prior to neutrophils stimulation with LPS (100 ng/mL). After 24 h, the plates were centrifuged, the supernatants were collected, and the cytokines release was determined by ELISA assay kits using a Synergy 4 microplate reader, according to the manufacturer’s instructions (BD Biosciences, San Jose, CA, USA). The effect on cytokine production was determined as percentage of released cytokines relative to the control without the investigated EOs. Urolithin A (a substance with known anti-inflammatory properties [48,49]) at concentrations of 12.5, 25, and 50 µM was used as a positive control.

### 2.9. Statistical Analyses

The results were calculated as a mean ± standard error of the mean (SEM) of the indicated number of experiments. One-way analysis of variance (ANOVA) with Tukey’s and Dunnet’s post hoc tests was used to evaluate the statistical significance that was set at *p* < 0.05.

## 3. Results

### 3.1. Yield and Chemical Composition of Essential Oils

The highest essential oil yield, as calculated on the basis of the weight of the dry fruits (triplicate experiments, percentage ± SEM), was determined for ajowan (7.23 ± 0.09%), followed by anise (3.13 ± 0.07%), caraway (2.83 ± 0.03%), and coriander (2.13 ± 0.09%). The phytochemical compositions of the four Apiaceae EOs assessed by GC are summarized in Table 1.

In the case of ajowan EO, up to 98.09% of the total constituents were identified, with thymol as the main compound (49.32%), followed by *γ*-terpinene (23.18%), and *m*-cymene (21.09%). From the total constituents, 98.76% were identified in coriander EO; linalool was the major detected compound (67.87%), alongside several minor constituents, such as *α*-pinene (8.13%), *γ*-terpinene (5.77%), camphor (3.82%), and geranyl acetate (3.71%). Carvone and limonene were the main compounds identified in caraway EO (55.40% and 42.74%, respectively), plus additional volatiles found in trace amounts, together making up to 99.02% of the total constituents. Anise EO was characterized by the presence of anethole as the major compound (90.01%), followed by germacrene (2.95%), estragole (1.28%), and other minor constituents, representing 98.48% of the total volatile fraction. The EOs were mainly characterized by the presence of monoterpenes (monoterpene hydrocarbons and oxygenated monoterpenes) in the case of ajowan, coriander, and caraway EOs; meanwhile, the anise EO comprised phenylpropanoids and sesquiterpene hydrocarbons.

### 3.2. Antifungal Susceptibility Results

The antifungal activity of Apiaceae EOs and their major constituents (thymol, linalool, carvone, limonene, anethole) was assessed using the microdilution method. The MIC and MFC values obtained for EO/main compounds against *T. rubrum* and *T. mentagrophytes* are shown in Table 2.

Ajowan EO exhibited the highest antifungal potential against the tested strains, with MICs of 256 mg/L. Coriander and caraway EOs showed a similar antifungal activity pattern, inhibiting the growth of both dermatophytes, with MIC values of 512 mg/L. Anise EO was the least active among the investigated EOs, displaying fungistatic effects at 1024 mg/L. With respect to the antidermatophytic activity of EOs, the main compounds, thymol, linalool, and carvone, acted similarly towards both *Trichophyton* strains, with MICs of 1024 mg/mL; meanwhile, limonene and anethole exhibited a lower degree of dermatophytic growth inhibition (MIC 2048 mg/L). Compared to the investigated EOs and their main constituents, terbinafine was highly active against dermatophytes (MIC 0.031 mg/L). Based on the MFC values, all EOs/main compounds were found to display a fungicidal activity against strains of *T. rubrum* and *T. mentagrophytes* (Table 2).

### 3.3. In Vitro Effects of Essential Oils/Main Constituents and Terbinafine Combinations against Dermatophytes

In order to evaluate the putative synergistic combinations of EOs and their main constituents with terbinafine, the checkerboard microtiter assay was performed. Considering their high sensitivity to terbinafine, in vitro experiments using *T. rubrum* and *T. mentagrophytes* strains were undertaken. The combinatorial effects of the investigated EOs/compounds with terbinafine are reported in Table 3. As revealed by the checkerboard testing, the binary associations of all the tested EOs with terbinafine were found to be synergistic against the *T. rubrum* strain, with FICI values of 0.26 (coriander and anise EOs), 0.28 (caraway EO), and 0.31 (ajowan EO), respectively. Moreover, EOs significantly potentiated the activity of terbinafine in combinatorial therapy, with a 4-fold reduction in their MIC, from 0.031 to 0.007 mg/L. In addition, the MIC of EOs significantly decreased in combination with terbinafine; a 64-fold reduction in the case of coriander and anise EOs, while MICs of caraway and ajowan EOs underwent a 32-fold and a 16-fold reduction, respectively. The main constituents of the EOs showed only additive effects in combination with terbinafine (FICI values range of 0.53–0.56). Similar additive interactions were displayed by the associations between the EOs/volatile compounds and terbinafine against *T. mentagrophytes*, with FICI values ranging from 0.56 to 0.75.

### 3.4. Effects on Cytokine Production in Human Neutrophils

Based on the MIC values obtained from the synergistic combinations with terbinafine, the four EOs (concentration range 6.25–25 mg/L) were next assessed for their ability to influence the viability of human neutrophils and the production of pro-inflammatory cytokines in LPS-stimulated neutrophils.

#### 3.4.1. Effects on Neutrophils Viability

**Figure 1:**
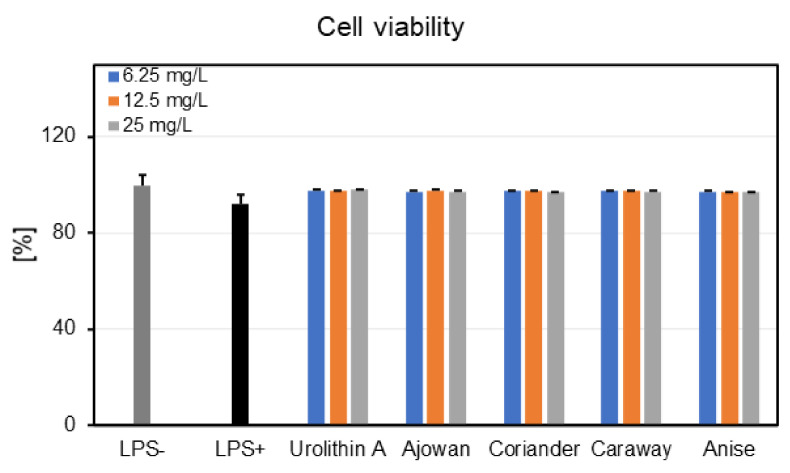
Neutrophils viability (%) following 24 h-treatment with essential oils of ajowan, coriander, caraway, and anise (6.25–25 mg/L), as well as urolithin A (12.5, 25 and 50 μM). Results are expressed as mean ± SEM of three separate experiments performed with cells isolated from four independent donors.

None of the tested EOs concentrations induced cytotoxic effects in human neutrophils as compared to negative control cells (LPS-) (Figure 1). For instance, at 25 mg/L, the cell viabilities ranged from 96.82% to 97.68%. In addition, the activation of neutrophils with LPS did not significantly reduce their viability (cell viability: 92.20%). Furthermore, the treatment with urolithin A, used as the positive control in the following cytokine production assays, did not display cytotoxic effects towards neutrophils (viability of neutrophils 97.62–98.06%). Meanwhile, Triton X-100 (0.1%) caused a very strong reduction in the cell viability, with only 1.63% viable cells (data not shown).

#### 3.4.2. Effects on Pro-Inflammatory Cytokine Secretion

**Figure 2:**
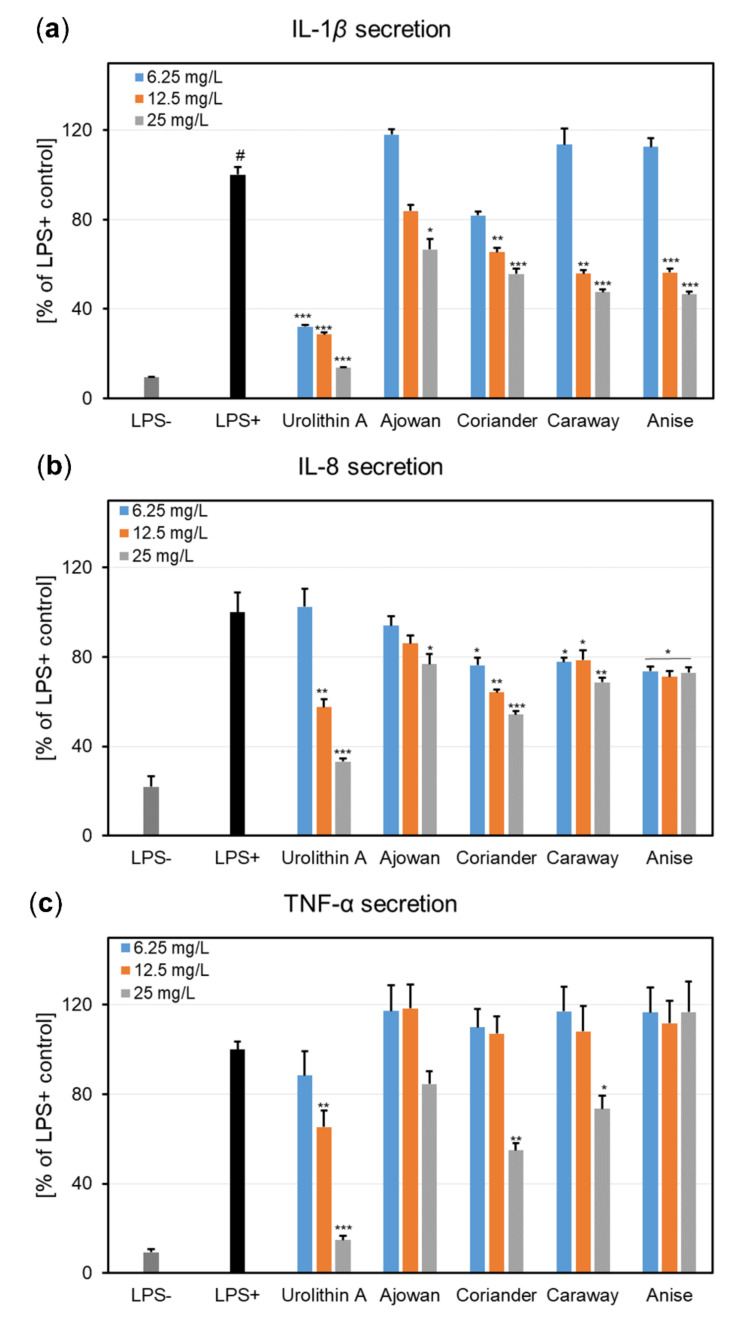
Effects of essential oils of ajowan, coriander, caraway, and anise (6.25–25 mg/L), and urolithin A (12.5, 25 and 50 μM) on IL-1*β* (**a**), IL-8 (**b**), and TNF-*α* (**c**) secretion in LPS-stimulated neutrophils. Results were expressed as mean ± SEM of three separate experiments performed with cells isolated from four independent donors. Statistical significance: # *p* < 0.001 compared to the non-stimulated control (LPS-); * *p* < 0.05, ** *p* < 0.01, *** *p* < 0.001 compared to the stimulated control (LPS+).

The effects of EOs on the release of pro-inflammatory cytokines, namely IL-1*β*, IL-8, and TNF-*α*, were assessed by ELISA in an ex vivo, LPS-stimulated neutrophil model (Figure 2).

When tested at non-cytotoxic concentrations (6.25, 12.5, and 25 mg/L), the four Apiaceae EOs exhibited a concentration-dependent reduction in IL-1*β* release (Figure 2a). At 25 mg/L, the EOs reduced the IL-1*β* levels to 66.64% (ajowan), 55.62% (coriander), 47.50% (caraway), and 46.49% (anise), when compared to LPS-stimulated neutrophils. Nevertheless, the EOs potency was lower when compared to urolithin A, which decreased the IL-1*β* production to 13.64% of the LPS+ control at 50 μM. The potential of EOs to act as IL-8 inhibitors is depicted in Figure 2b. Out of the four tested EOs, the coriander EO was the most active, interfering with IL-8 release in a concentration-dependent manner; the IL-8 levels at 25 mg/L were decreased to 54.15% of LPS+ control. A similar effect was observed for urolithin A at 25 μM (57.61% of LPS+ control). Ajowan EO was found to slightly inhibit the IL-8 production at the highest tested concentration (76.74% of LPS+ control). The effects of the preincubation of human neutrophils with Apiaceae EOs upon TNF-*α* release after stimulation with LPS is presented in Figure 2c. At the highest tested concentration (25 mg/L), only coriander and caraway EOs were able to produce a slight to moderate decrease in TNF-*α* levels (54.91% and 73.42% of LPS+ control, respectively), whereas the other two EOs were inactive over the tested concentration range. In contrast, urolithin A produced strong TNF-*α* inhibitory effects, especially at 50 μM (14.77% of LPS+ control).

## 4. Discussion

Even though combinatorial strategies including antifungal drugs and EOs are already recommended to improve monotherapy, the literature reports regarding terbinafine’s association with these plant-derived products are scarce [50,51,52]. Our study aimed to identify putative synergistic combinations of terbinafine using the selected Apiaceae EOs (ajowan, coriander, caraway, anise) and their major compounds (thymol, linalool, carvone, limonene, and anethole) against the main causative agents of dermatophytosis, *T. rubrum,* and *T. mentagrophytes*.

Therefore, the antifungal potential was first tested using broth microdilution method. According to de Oliveira Lima et al. [53], the antimicrobial potential of plant extracts is correlated with their MIC values, as follows: strong activity (MIC 50–500 mg/L), moderate activity (500 mg/L > MIC < 1500 mg/L), and weak activity (MIC > 1500 mg/L). Thus, ajowan EO was highly active against all tested fungi (MIC 256 mg/L), while coriander, caraway, and anise EOs displayed a moderate activity (MIC > 500 mg/L). Among the tested volatile compounds, thymol, linalool, and carvone also had a moderate antifungal potential (MIC 1024 mg/L); meanwhile, limonene and anethole possessed a low antimicrobial activity (MIC 2048 mg/L). Compared to the parent EOs, the antifungal potential of their main constituents was lower, suggesting that other minor compounds might contribute, via phytosynergic interactions, to the overall bioactivity of EOs. Even though several studies assessed the antidermatophytic potential of investigated Apiaceae EOs and their major components [54,55,56,57,58,59], a direct comparison between the MICs obtained in the present study and the literature data is hampered as a consequence of the implementation of different protocols (e.g., obtaining the serial dilutions, fungi growth phase and vitality, inoculum volume, culture medium and pH, temperature, and incubation time). In addition, our results showed that EOs and their main compounds acted as fungicides against both *T. rubrum* and *T. mentagrophytes* strains. This is of the highest importance in clinical settings of recurrent and multi-drug resistant dermatophytoses, as the use of fungistatic agents is commonly associated with fungal resistance [10,14].

Still, even if the antifungal effects of the investigated EOs do not justify their use as stand-alone antimicrobial agents, it is noteworthy that, at significantly lower subinhibitory concentrations (1/16xMIC–1/64xMIC), EOs induced a 4-fold enhancement of terbinafine activity against *T. rubrum*. The observed synergistic effects may be due to the ability of EOs to facilitate terbinafine entry into the cell with an enhancement in its inhibitory effects towards ergosterol biosynthesis. The Apiaceae EOs investigated in our study mostly comprised monoterpene hydrocarbons and oxygenated monoterpenes (ajowan, coriander, and caraway EOs), and phenylpropanoids (anise EO). It is known that such compounds are lipophilic and have a small molecular size which enable their passive diffusion through fungi membranes [16]. Volatile compounds exert their fungicidal activity through different mechanisms of action that include, among others, the perturbation of the lipid membrane organization [60] followed by the increased permeability of the fungal cell wall/membrane, the extravasation of cell constituents, the inhibition of ergosterol synthesis, and cell lysis [15,23]. Moreover, it was reported that several main constituents of the Apiaceae EOs included in our study acted on the virulence factors and resistance mechanisms of *T. rubrum.* For example, Ponte et al. [54] showed that linalool, the major compound of coriander EO, acts as an efflux pump inhibitor and is able to increase the susceptibility to azoles of *Trichophyton* multi-drug resistant strains. Thymol, the main constituent of ajowan EO, enhanced fluconazole efficacy against the clinical isolates of *T. rubrum* by inhibiting the activity of proteinases (elastase and keratinase) that contribute to fungal virulence [61]. Furthermore, thymol displayed an inhibitory activity towards efflux pumps, which are one of the fungal resistance mechanisms [62]. Obaid et al. [63] demonstrated that anethole, identified as the main compound in the anise EO, was able to down-regulate the keratinase gene expression in *T. rubrum* strains.

Herein, we reported for the first time the synergistic combinatorial effects of terbinafine with EOs derived from the fruits of ajowan, coriander, caraway, and anise. As of now, the literature data on terbinafine’s synergistic associations with EOs is scarce. To the best of our knowledge, only two studies reported on the ability of *Lavandula luisieri* and *Schinus lentiscifolius*-derived EOs to enhance terbinafine’s activity against the clinical isolates of *T. rubrum* [49,50]. One must note that these species grow spontaneously in specific habitats: *L. luisieri* is endemic to the Iberic Peninsula, while *S. lentiscifolius* grows in southern Brazil, therefore representing a limited supply that could hamper their industrial use [51,52]. Our study included species which are commonly used as spices and are cultivated on a large scale [23]. Thus, the plant material for the essential oil isolation is readily available and can be constantly supplied to the pharmaceutical industry. In addition, the development of Apiaceae EOs-based formulations should also consider the quality and standardization of EOs. It is known that the chemical variability of EOs is mainly due to the plant phenotype, pedoclimatic conditions, harvesting and storage conditions. To overcome batch-to-batch variability, valuable cultivars might supply a plant material with a constant chemical profile, thus a reliable source for the pharmaceutical industry. Our results are of utmost importance in the case of recalcitrant dermatophytoses when higher doses of terbinafine and long-term treatment are required, which in turn entail hepatotoxic events and a poor adherence to therapy regimens [6]. Therefore, the association with EOs could minimize the side effects of the dose-related toxicity of terbinafine and increase the efficacy of the treatment compared to monotherapy. In addition, such combinations could prove useful in the topical treatment of onychomycosis, as EOs are blends of molecules with a low molecular weight that act as nail permeation enhancers for terbinafine, thus ensuring a a higher efficacy of the treatment [18,64,65]. Moreover, considering that EOs act concurrently towards different microbial targets due to their multicomponent nature, the selection of resistant fungal strains can be overcome [16,66].

Based on the MIC values obtained from the synergistic combinations with terbinafine (Table 3), Apiaceae EOs were evaluated for their ability to influence the secretion of pro-inflammatory cytokines in ex vivo-stimulated human neutrophils. Human neutrophils are primarily involved in the host defense mechanism against *T. rubrum*, as both clinical setups and animal model experiments revealed a dense infiltration of neutrophils in infected areas [67]. Following recruitment from the bloodstream, neutrophils’ activation and responses to a fungi attack include: phagocytosis, an oxidative burst by reactive oxygen species production, the secretion of proteases, the release of extracellular traps, the secretion of pro-inflammatory cytokines (e.g., IL-1*β*, IL-6, IL-8, and TNF-*α*), chemokines, and growth factors [1,68,69]. However, the prolonged activation of neutrophils hinders the resolution of infection, sustaining a chronic inflammation which in turn can contribute to colonization of the neighboring host tissue [70]. Therefore, therapeutic alternatives that combine selective antifungal and anti-inflammatory activities should also be considered to tune the fine balance between pro- and anti-inflammatory signals in human–host fungi interactions.

First, the potential deleterious effects of Apiaceae EOs were investigated towards human neutrophils, obtained ex vivo from healthy volunteers. EOs did not affect neutrophils viability, as no cytotoxic effects were recorded at the tested concentrations (6.25–25 mg/L) (Figure 1), thus proving the safety of EOs for pharmaceutical and cosmetic use. Moreover, neutrophils showed a good viability and released functional, pro-inflammatory cytokines following LPS-stimulation, such as IL-1*β*, IL-8, and TNF-*α* (Figure 2). Our results revealed that EOs inhibited, in different degrees of potency, the production of cytokines in LPS-stimulated neutrophils. It was observed that the inhibition of IL-1*β* release by EOs was concentration-dependent (Figure 2a), with the anise EO displaying the most potent inhibitory activity, followed by caraway, coriander, and ajowan EOs. Regarding IL-8 and TNF-*α*, coriander EO proved the highest ability to lower their production in stimulated neutrophils (Figure 2b,c).

To the best of our knowledge, we reported herein for the first time the inhibitory activity of EOs isolated from the fruits of ajowan, coriander, caraway, and anise, towards IL-1*β*, IL-8, and TNF-*α* secretion in an ex vivo human neutrophils model. Our results are in accordance with previously published data regarding the influence of the investigated Apiaceae EOs and their main compounds on pro-inflammatory cytokine production. Thus, ajowan EO was shown to possess anti-inflammatory effects as assessed by in vitro studies on murine macrophage cells, by inhibiting cyclooxygenase-2, inducible nitric oxide synthase, and heme oxygenase-1 [71]. Coriander EO exhibited anti-inflammatory effects in an *in vivo* model of colitis as determined by histo-pathologic evaluation and myeloperoxidase activity [72]; meanwhile, its main constituent linalool was found to inhibit the production of pro-inflammatory cytokines (e.g., TNF-*α*, IL-1*β*, IL-6, IL-8) in both in vitro and in vivo settings [73,74]. Caraway EO has shown a protective activity in animal models of colitis and renal injury by the inhibition of cytokine production and the induction of endogenous antioxidant enzymatic systems [75,76]; its beneficial effects are mostly due to limonene and carvone as revealed by various in vitro and in vivo studies [77,78,79]. As for anise EO, it has proven in vitro anti-inflammatory propensities by the inhibition of IL-1*β* and IL-8 secretion in a bronchial epithelial cell line [80], while its main constituent, anethole was shown to decrease IL-6 and TNF-*α* serum concentrations in an animal model of chronic and obstructive pulmonary disease [81].

Correlating the results from the checkerboard testing, where Apiaceae EOs showed potent synergistic combinations with terbinafine, alongside the inhibition of pro-inflammatory cytokines release, we can conclude that the further development of formulations including the investigated EOs and terbinafine are justified.

Considering the lipophilic nature of EOs components, their high volatility and chemical instability, the encapsulation in nanoformulated delivery systems could be an approach to overcome such limitations and boost the antidermatophytic activity of EOs [15,22,82]. The additional data regarding their skin and nail permeation, elucidation of their mechanism of activity, and whether in vitro results translate into similar outcomes in in vivo models, are in high demand. In addition, the development of pharmaceutical formulations should consider several parameters, including the stability of such combinations, their pharmacokinetic profiles, and their safety upon administration. Moreover, the mode of administration significantly influences the availability of EOs at the infection site. Thus, a topical use of Apiaceae EOs–terbinafine combinations is readily envisioned.

## 5. Conclusions

The investigated combinations of Apiaceae EOs/main compounds and terbinafine showed synergistic and additive effects against *T. rubrum* and *T. mentagrophytes*, thus reducing the active concentration of the antifungal drug when used together. Therefore, the use of such mixtures might provide a better outcome than monotherapy in terms of side effects and toxicity, but also in decreasing the emergence of resistant strains. Based on their antifungal and anti-inflammatory activities, these combinatorial strategies could be complementary to conventional therapy by alleviating symptoms, aiding the healing process, and preventing dermatophytosis dissemination. Overall, our study highlights the putative use of synergistic combinations of terbinafine and investigated Apiaceae EOs (ajowan, coriander, caraway, and anise) as a starting point for the development of novel topical formulations for *T. rubrum*-related dermatophytosis.

## Figures and Tables

**Table 1:** Main constituents of essential oils isolated from Apiaceae fruits.

RI *	Compound	Ajowan (%)	Coriander (%)	Caraway (%)	Anise (%)
	*Monoterpene hydrocarbons*				
928	*α*-Thujene	0.54	-	-	-
931	*α*-Pinene	0.27	8.13	0.03	-
943	Camphene	-	1.02	-	-
960	*β*-Phellandrene	-	-	0.06	-
965	Sabinene	-	0.40	-	-
970	*β*-Pinene	2.48	0.72	0.02	-
979	Myrcene	0.62	1.80	0.41	-
1016	*m*-Cymene	21.09	0.92	-	-
1023	*β*-Thujene	0.18	-	-	-
1033	Limonene	-	2.36	42.74	-
1047	*γ*-Terpinene	23.18	5.77	-	-
1088	*α*-Terpinolene	-	0.57	-	-
	*Oxygenated monoterpenes*				
1104	Linalool	-	67.87	-	0.32
1150	Terpinen-4-ol	0.17	-	-	-
1157	Camphor	-	3.82	-	-
1230	Geraniol	-	1.67	-	-
1238	Carvone	-	-	55.40	0.32
1242	Perillaldehyde	-	-	0.24	-
1275	Thymol	49.32	-	-	-
1278	Carvacrol	0.24	-	-	-
1323	Methyl geranate	-	-	0.12	-
1360	Geranyl acetate	-	3.71	-	-
	*Sesquiterpene hydrocarbons*				
1480	Germacrene D	-	-	-	2.95
1490	Zingiberene	-	-	-	0.29
1499	*β*-Himachalene	-	-	-	0.14
1505	*β*-Bisabolene	-	-	-	0.26
1542	*σ*-Himachalene	-	-	-	0.19
	*Oxygenated sesquiterpenes*				
1601	Spathulenol	-	-	-	0.09
	*Phenylpropanoids*				
1180	Estragole	-	-	-	1.94
1249	*p*-Anisaldehyde	-	-	-	0.78
1280	Anethole	-	-	-	90.01
1831	Isoeugenyl acetate	-	-	-	1.19
	*Monoterpene hydrocarbons*	48.36	21.69	43.26	-
	*Oxygenated monoterpenes*	49.73	77.07	55.76	0.64
	*Sesquiterpene hydrocarbons*	-	-	-	3.83
	*Oxygenated sesquiterpenes*	-	-	-	0.09
	*Phenylpropanoids*	-	-	-	93.92
	**Total**	**98.09**	**98.76**	**99.02**	**98.48**

* Retention indices relative to a series of C8–C20 *n*-alkanes calculated on TRB-5MS column. “-” not detected.

**Table 2:** Antifungal activity (MIC, MFC expressed as mg/L) of Apiaceae essential oils and their main constituents.

Sample/Positive Control	*Trichophyton rubrum* ATCC 28188		*Trichophyton mentagrophytes* ATCC 9533	
	**MIC**	**MFC**	**MIC**	**MFC**
**Ajowan essential oil**	256	512	256	512
**Coriander essential oil**	512	1024	512	1024
**Caraway essential oil**	512	512	512	512
**Anise essential oil**	1024	2048	1024	2048
**Thymol**	1024	1024	1024	1024
**Linalool**	1024	1024	1024	2048
**Carvone**	1024	2048	1024	2048
**Limonene**	2048	2048	2048	2048
**Anethole**	2048	2048	2048	2048
**Terbinafine**	0.031	0.031	0.031	0.031

MIC = minimum inhibitory concentration; MFC = minimum fungicidal concentration.

**Table 3:** Combinatorial effects (FIC and FICI values) between Apiaceae essential oils/main constituents and terbinafine assessed by checkerboard method.

Combination	*Trichophyton rubrum* ATCC 28188			INT	*Trichophyton mentagrophytes* ATCC 9533			INT
	MIC ^*^_in combination_ (mg/L)	FIC	FICI		MIC ^*^_in combination_ (mg/L)	FIC	FICI	
**Ajowan essential oil**	16	0.06	0.31	**S**	16	0.06	0.56	**Ad**
**Terbinafine**	0.007	0.25			0.015	0.50		
**Coriander essential oil**	8	0.01	0.26	**S**	32	0.06	0.56	**Ad**
**Terbinafine**	0.007	0.25			0.015	0.50		
**Caraway essential oil**	16	0.03	0.28	**S**	64	0.12	0.62	**Ad**
**Terbinafine**	0.007	0.25			0.015	0.50		
**Anise essential oil**	16	0.01	0.26	**S**	128	0.12	0.62	**Ad**
**Terbinafine**	0.007	0.25			0.015	0.50		
**Thymol**	64	0.06	0.56	**Ad**	128	0.12	0.62	**Ad**
**Terbinafine**	0.015	0.5			0.015	0.5		
**Linalool**	32	0.03	0.53	**Ad**	256	0.25	0.75	**Ad**
**Terbinafine**	0.015	0.5			0.015	0.5		
**Carvone**	32	0.03	0.53	**Ad**	256	0.25	0.75	**Ad**
**Terbinafine**	0.015	0.5			0.015	0.5		
**Limonene**	64	0.03	0.53	**Ad**	256	0.12	0.62	**Ad**
**Terbinafine**	0.015	0.5			0.015	0.5		
**Anethole**	128	0.06	0.56	**Ad**	512	0.25	0.75	**Ad**
**Terbinafine**	0.015	0.5			0.015	0.5		

Ad—addition; FIC—fractional inhibitory concentration; FICI—fractional inhibitory concentration index; INT—interpretation; S—synergism. * MIC of tested agent in most effective combination.

## Data Availability

Not applicable.

## References

[1] Burstein V.L., Beccacece I., Guasconi L., Mena C.J., Cervi L., Chiapello L.S. (2020). Skin immunity to dermatophytes: From experimental infection models to human disease. Front. Immunol..

[2] Gnat S., Łagowski D., Nowakiewicz A. (2020). Major challenges and perspectives in the diagnostics and treatment of dermatophyte infections. J. Appl. Microbiol..

[3] de Hoog G.S., Dukik K., Monod M., Packeu A., Stubbe D., Hendrickx M., Kupsch C., Stielow J.B., Freeke J., Göker M. (2017). Toward a novel multilocus phylogenetic taxonomy for the dermatophytes. Mycopathologia.

[4] Nenoff P., Verma S.B., Vasani R., Burmester A., Hipler U.C., Wittig F., Krüger C., Nenoff K., Wiegand C., Saraswat A. (2019). The current Indian epidemic of superficial dermatophytosis due to *Trichophyton mentagrophytes*—A molecular study. Mycoses.

[5] Zhan P., Liang G., Liu W., Bouchara J.P., Nenoff P., Gupta A.K., Chaturvedi V. (2021). Dermatophytes and dermatophytic infections worldwide. Dermatophytes and Dermatophytoses.

[6] Gupta A.K., Renaud H.J., Quinlan E.M., Shear N.H., Piguet V. (2021). The growing problem of antifungal resistance in onychomycosis and other superficial mycoses. Am. J. Clin. Dermatol..

[7] Monod M., Feuermann M., Yamada T., Bouchara J.P., Nenoff P., Gupta A.K., Chaturvedi V. (2021). Terbinafine and itraconazole resistance in dermatophytes. Dermatophytes and Dermatophytoses.

[8] Kramer O., Albrecht J. (2017). Clinical presentation of terbinafine-induced severe liver injury and the value of laboratory monitoring: A Critically Appraised Topic. Brit. J. Dermatol..

[9] Durdu M., Ilkit M., Tamadon Y., Tolooe A., Rafati H., Seyedmousavi S. (2017). Topical and systemic antifungals in dermatology practice. Expert Rev. Clin. Pharmacol..

[10] Gupta A.K., Venkataraman M., Quinlan E.M., Bouchara J.P., Nenoff P., Gupta A.K., Chaturvedi V. (2021). New antifungal agents and new formulations against dermatophytes. Dermatophytes and Dermatophytoses.

[11] Elewski B., Ghannoum M., Mayser P., Gupta A., Korting H.C., Shouey R., Baker D., Rich P., Ling M., Hugot S. (2013). Efficacy, safety and tolerability of topical terbinafine nail solution in patients with mild to moderate toenail onychomycosis: Results from three randomized studies using double blind vehicle controlled and open label active controlled designs. J. Eur. Acad. Dermatol. Venereol..

[12] Saunte D.M., Hare R.K., Jørgensen K.M., Jørgensen R., deleuran M., Zachariae C.O., Thomsen S.F., Bjørnskov-Halkier L., Kofoed K., Arendrup M.C. (2019). Emerging terbinafine resistance in *Trichophyton*: Clinical characteristics, squalene epoxidase gene mutations, and a reliable EUCAST method for detection. Antimicrob. Agents Chemother..

[13] Khurana A., Sardana K., Chowdhary A. (2019). Antifungal resistance in dermatophytes: Recent trends and therapeutic implications. Fungal Genet. Biol..

[14] Lopes G., Pinto E., Salgueiro L. (2017). Natural products: An alternative to conventional therapy for dermatophytosis?. Mycopathologia.

[15] Brescini L., Fioriti S., Morroni G., Barchiesi F. (2021). Antifungal combinations in dermatophytes. J. Fungi.

[16] Zuzarte M., Lopes G., Pinto E., Salgueiro L., Bouchara J.P., Nenoff P., Gupta A.K., Chaturvedi V. (2021). Are natural products an alternative therapy for dermatophytosis?. Dermatophytes and Dermatophytoses.

[17] Van Vuuren S., Viljoen A. (2011). Plant-based antimicrobial studies–methods and approaches to study the interaction between natural products. Planta Med..

[18] Adorjan B., Buchbauer G. (2010). Biological properties of essential oils: An updated review. Flavour Fragr. J..

[19] Hammer K.A., Carson C.F., Thormar H. (2011). Antibacterial and antifungal activities of essential oils. Lipids and Essential Oils as Antimicrobial Agents.

[20] Bakkali F., Averbeck S., Averbeck D., Idaomar M. (2008). Biological effects of essential oils—A review. Food Chem. Toxicol..

[21] Bruneton J. (2008). Pharmacognosy, Phytochemistry, Medicinal Plants.

[22] Trifan A., Luca S.V., Greige-Gerges H., Miron A., Gille E., Aprotosoaie A.C. (2020). Recent advances in tackling microbial multidrug resistance with essential oils: Combinatorial and nano-based strategies. Crit. Rev. Microbiol..

[23] Lopes A.I., Tavaria F.K., Pintado M.E. (2020). Conventional and natural compounds for the treatment of dermatophytosis. Med. Mycol..

[24] D’agostino M., Tesse N., Frippiat J.P., Machouart M., debourgogne A. (2019). Essential oils and their natural active compounds presenting antifungal properties. Molecules.

[25] Simpson M.G. (2019). Plant Systematics.

[26] Aćimović M., Mérillon J.M.., Ramawat K. (2017). Nutraceutical potential of Apiaceae. Bioactive Molecules in Food. Reference Series in Phytochemistry.

[27] Heinrich M., Williamson E.M., Gibbons S., Barnes J., Prieto-Garcia J. (2012). Fundamentals of Pharmacognosy and Phytotherapy.

[28] Widelski J., Graikou K., Ganos C., Skalicka-Wozniak K., Chinou I. (2021). Volatiles from selected Apiaceae species cultivated in Poland—Antimicrobial activities. Processes.

[29] Khalil N., Ashour M., Fikry S., Singab A.N., Salama O. (2018). Chemical composition and antimicrobial activity of the essential oils of selected Apiaceous fruits. FJPS.

[30] Elshafie H.S., Camele I. (2017). An overview of the biological effects of some mediterranean essential oils on human health. BioMed Res. Int..

[31] Sayed-Ahmad B., Talou T., Saad Z., Hijazi A., Merah O. (2017). The Apiaceae: Ethnomedicinal family as source for industrial uses. Ind. Crops Prod..

[32] Trifan A., Bostănaru A.-C., Luca S.V., Grădinaru A.C., Jităreanu A., Aprotosoaie A.C., Miron A., Cioancă O., Hăncianu M., Ochiuz L. (2020). Antifungal potential of *Pimpinella anisum, Carum carvi* and *Coriandrum sativum* extracts. A comparative study with focus on the phenolic composition. Farmacia.

[33] Grădinaru A., Trifan A., Şpac A., Brebu M., Miron A., Aprotosoaie A. (2018). Antibacterial activity of traditional spices against lower respiratory tract pathogens: Combinatorial effects of *Trachyspermum ammi* essential oil with conventional antibiotics. Lett. Appl. Microbiol..

[34] Trifan A., Aprotosoaie A.C., Cioanca O., Hancianu M., Jitareanu A., Gille E., Miron A. (2016). Antioxidant activity of essential oil from *Carum carvi* L. cultivated in North-eastern Romania. Med.-Surg. J..

[35] Trifan A., Miron A., Aprotosoaie A.C., Hancianu M., Cioanca O., Gille E., Stanescu U. (2013). Phytotoxicity assessment of polyphenolic extracts from *Carum carvi* L. fruits. Farmacia.

[36] Trifan A., Aprotosoaie A.C., Şpac A., Hăncianu M., Miron A., Stănescu U. (2012). Contributions to the chemical study of the essential oil isolated from coriander (Omagiu cultivar) fruits. Farmacia.

[37] Jain N., Sharma M., Joshi S., Kaushik U. (2018). Chemical composition, toxicity and antidermatophytic activity of essential oil of *Trachyspermum ammi*. Indian J. Pharm. Sci..

[38] Swamy M.K., Akhtar M.S., Sinniah U.R. (2016). Antimicrobial properties of plant essential oils against human pathogens and their mode of action: An updated review. Evid.-Based Complementary Altern. Med..

[39] Laribi B., Kouki K., M’Hamdi M., Bettaieb T. (2015). Coriander (*Coriandrum sativum* L.) and its bioactive constituents. Fitoterapia.

[40] Bairwa R., Sodha R., Rajawat B. (2012). *Trachyspermum* *ammi*. Pharmacogn. Rev..

[41] Navarro-Rocha J., Andrés M.F., Díaz C.E., Burillo J., González-Coloma A. (2020). Composition and biocidal properties of essential oil from pre-domesticated Spanish *Satureja montana*. Ind. Crops Prod..

[42] NIST Chemistry WebBook NIST Standard Reference Database Number 69. https://webbook.nist.gov/chemistry.

[43] Arendrup M., Meletiadis J., Mouton J., Lagrou K., Hamal P., Guinea J. Eucast Definitive Document E DEF. 9.3.1. Method for the Determination of Broth Dilution Minimum Inhibitory Concentrations of Antifungal Agents for Conidia Forming Moulds. London: European Committee on Antimicrobial Susceptibility Testing. https://www.eucast.org/ast_of_fungi/.

[44] Markantonatou A.-M., Samaras K., Zachrou E., Vyzantiadis T.-A. (2020). Comparison of four methods for the in vitro susceptibility testing of dermatophytes. Front. Microbiol..

[45] Verma P., Schwalbe R., Steele-Moore L., Goodwin A.C. (2007). Methods for determining bactericidal activity and antimicrobial interactions: Synergy testing, time-kill curves, and population analysis. Antimicrobial Susceptibility Testing Protocols.

[46] Czerwińska M.E., Dudek M.K., Pawłowska K.A., Pruś A., Ziaja M., Granica S. (2018). The influence of procyanidins isolated from small-leaved lime flowers (*Tilia cordata* Mill.) on human neutrophils. Fitoterapia.

[47] Trifan A., Skalicka-Woźniak K., Granica S., Czerwińska M.E., Kruk A., Marcourt L., Wolfender J.-L., Wolfram E., Esslinger N., Grubelnik A. (2020). *Symphytum officinale* L.: Liquid-liquid chromatography isolation of caffeic acid oligomers and evaluation of their influence on pro-inflammatory cytokine release in LPS-stimulated neutrophils. J. Ethnopharmacol..

[48] Rønning S.B., Voldvik V., Bergum S.K., Aaby K., Borge G.I.A. (2020). Ellagic acid and urolithin A modulate the immune response in LPS-stimulated U937 monocytic cells and THP-1 differentiated macrophages. Food Funct..

[49] Fu X., Gong L.F., Wu Y.F., Lin Z., Jiang B.J., Wu L., Yu K.H. (2019). Urolithin A targets the PI3K/Akt/NF-κB pathways and prevents IL-1β-induced inflammatory response in human osteoarthritis: In *vitro* and in vivo studies. Food Funct..

[50] Danielli L.J., Pippi B., Soares K.D., Duarte J.A., Maciel A.J., Machado M.M., Oliveira L.F.S., Bordignon S.A., Fuentefria A.M., Apel M.A. (2017). Chemosensitization of filamentous fungi to antifungal agents using *Nectandra* Rol. ex Rottb. species essential oils. Ind. Crops Prod..

[51] Danielli L.J., Pippi B., Duarte J.A., Maciel A.J., Lopes W., Machado M.M., Oliveira L.F.S., Vainstein M.H., Teixeira M.L., Bordignon S.A. (2018). Antifungal mechanism of action of *Schinus lentiscifolius* Marchand essential oil and its synergistic effect in vitro with terbinafine and ciclopirox against dermatophytes. J. Pharm. Pharmacol..

[52] Dias N., Dias M., Cavaleiro C., Sousa M., Lima N., Machado M. (2017). Oxygenated monoterpenes-rich volatile oils as potential antifungal agents for dermatophytes. Nat. Prod. Res..

[53] de Oliveira Lima M., de Medeiros A.A., Silva K.S., Cardoso G., de Oliveira Lima E., de Oliveira Pereira F. (2017). Investigation of the antifungal potential of linalool against clinical isolates of fluconazole resistant *Trichophyton rubrum*. J. Mycol. Med..

[54] Ponte H.A.S., Lima M.I.D.O., Lima E.D.O., Pereira F.D.O. (2020). Linalool modulates dermatophyte susceptibility to azole drugs. Med. Mycol..

[55] Jain N., Sharma M. (2020). Inhibitory effect of some selected essential oil terpenes on fungi causing superficial infection in human beings. J. Essent. Oil Bear. Plants.

[56] Obaid A.J., Al-Janabi J.K.A., Taj-Aldin W.R. (2017). Chemical composition and bioactivity characteristics of *Pimpinella anisum* essential oil against *Trichophyton rubrum*. J. Glob. Pharma Technol..

[57] Pinto E., Gonçalves M.-J., Cavaleiro C., Salgueiro L. (2017). Antifungal activity of *Thapsia villosa* essential oil against C*andida, Cryptococcus, Malassezia, Aspergillus* and dermatophyte species. Molecules.

[58] Inouye S., Uchida K., Abe S. (2006). Vapor activity of 72 essential oils against a *Trichophyton mentagrophytes*. J. Infect. Chemother..

[59] Lim S., Shin S.-W. (2007). Synergism in antifungal activity against *Candida* and *Trichophyton* species in combination with the essential oil of *Coriandrum sativum* L. and antibiotics. Nat. Prod. Sci..

[60] Gharib R., Auezova L., Charcosset C., Greige-Gerges H. (2018). Effect of a series of essential oil molecules on DPPC membrane fluidity: A biophysical study. J. Iran. Chem. Soc..

[61] Khan M.S.A., Ahmad I., Cameotra S.S. (2014). *Carum copticum* and *Thymus vulgaris* oils inhibit virulence in *Trichophyton rubrum* and *Aspergillus* spp.. Braz. J. Microbiol..

[62] de Melo J.O., Bitencourt T.A., Fachin A.L., Cruz E.M.O., de Jesus H.C.R., Alves P.B., de Fátima Arrigoni-Blank M., de Castro Franca S., Beleboni R.O., Fernandes R.P.M. (2013). Antidermatophytic and antileishmanial activities of essential oils from *Lippia gracilis* Schauer genotypes. Acta Trop..

[63] Obaid A.J., Al-Janabi J., Taj-Aldin W.R. (2020). Bioactivities of anethole, astragalin and cryptochlorogenic acid extracted from anise oil and *Moringa oleifera* on the keratinase gene expression of *Trichophyton rubrum*. J Pure Appl. Microbiol..

[64] Flores F.C., Beck R.C., Da Silva C.D.B. (2016). Essential oils for treatment for onychomycosis: A mini-review. Mycopathologia.

[65] Vörös-Horváth B., Das S., Salem A., Nagy S., Böszörményi A., Kőszegi T., Pál S., Széchenyi A. (2020). Formulation of tioconazole and *Melaleuca alternifolia* essential oil pickering emulsions for onychomycosis topical treatment. Molecules.

[66] Christenson J.K., Peterson G.M., Naunton M., Bushell M., Kosari S., Baby K.E., Thomas J. (2018). Challenges and opportunities in the management of onychomycosis. J. Fungi.

[67] Calderon R., Hay R. (1987). Fungicidal activity of human neutrophils and monocytes on dermatophyte fungi, *Trichophyton quinckeanum* and *Trichophyton rubrum*. Immunology.

[68] Van der Linden M., Meyaard L. (2016). Fine-tuning neutrophil activation: Strategies and consequences. Immunol. Lett..

[69] Zhao G., Usui M.L., Lippman S.I., James G.A., Stewart P.S., Fleckman P., Olerud J.E. (2013). Biofilms and inflammation in chronic wounds. Adv. Wound Care.

[70] Romani L. (2011). Immunity to fungal infections. Nat. Rev. Immunol..

[71] Bahuguna A., Ramalingam S., Arumugam A., Natarajan D., Kim M. (2020). Molecular and *in silico* evidences explain the anti-inflammatory effect of *Trachyspermum ammi* essential oil in lipopolysaccharide induced macrophages. Process Biochem..

[72] Heidari B., Sajjadi S.E., Minaiyan M. (2016). Effect of *Coriandrum sativum* hydroalcoholic extract and its essential oil on acetic acid-induced acute colitis in rats. Avicenna J. Phytomed..

[73] Ma J., Xu H., Wu J., Qu C., Sun F., Xu S. (2015). Linalool inhibits cigarette smoke-induced lung inflammation by inhibiting NF-κB activation. Int. Immunopharmacol..

[74] Huo M., Cui X., Xue J., Chi G., Gao R., deng X., Guan S., Wei J., Soromou L.W., Feng H. (2013). Anti-inflammatory effects of linalool in RAW 264.7 macrophages and lipopolysaccharide-induced lung injury model. J. Surg. Res..

[75] Keshavarz A., Minaiyan M., Ghannadi A., Mahzouni P. (2013). Effects of *Carum carvi* L.(caraway) extract and essential oil on TNBS-induced colitis in rats. Res. Pharm. Sci..

[76] Dadkhah A., Fatemi F. (2011). Heart and kidney oxidative stress status in septic rats treated with caraway extracts. Pharm. Biol..

[77] Zhao M., Du J. (2020). Anti-inflammatory and protective effects of D-carvone on lipopolysaccharide (LPS)-induced acute lung injury in mice. J. King Saud Univ. Sci..

[78] Vieira A.J., Beserra F.P., Souza M., Totti B., Rozza A. (2018). Limonene: Aroma of innovation in health and disease. Chem. Biol. Interact..

[79] Andrade L.N., de Sousa D.P. (2013). A review on anti-inflammatory activity of monoterpenes. Molecules.

[80] Iannarelli R., Marinelli O., Morelli M.B., Santoni G., Amantini C., Nabissi M., Maggi F. (2018). Aniseed (*Pimpinella anisum* L.) essential oil reduces pro-inflammatory cytokines and stimulates mucus secretion in primary airway bronchial and tracheal epithelial cell lines. Ind. Crops Prod..

[81] Kim K.Y., Lee H.S., Seol G.H. (2017). Anti-inflammatory effects of trans-anethole in a mouse model of chronic obstructive pulmonary disease. Biomed. Pharmacother..

[82] Sebaaly C., Trifan A., Sieniawska E., Greige-Gerges H. (2021). Chitosan-coating effect on the characteristics of liposomes: A focus on bioactive compounds and essential oils: A review. Processes.

